# Clinical outcomes among children with primary nephrogenic diabetes insipidus

**DOI:** 10.1093/ckj/sfaf303

**Published:** 2025-10-01

**Authors:** Seon Hee Lim, Jin-Soon Suh, Ji Hyun Kim, Seung Jae Lee, Naye Choi, Ji Yeon Song, Eujin Park, Hee Sun Baek, Hyun Kyung Lee, Se Jin Park, Min Ji Park, Min Hyun Cho, Eun Mi Yang, Hee Gyung Kang, Yo Han Ahn

**Affiliations:** Department of Pediatrics, Pusan National University Yangsan Children’s Hospital and School of Medicine, Yangsan, South Korea; Department of Pediatrics, Bucheon St. Mary’s Hospital, College of Medicine, The Catholic University of Korea, Seoul, South Korea; Department of Pediatrics, Seoul National University Bundang Hospital, Seongnam, South Korea; Department of Pediatrics, Seoul National University College of Medicine, Seoul, South Korea; Department of Pediatrics, Seoul National University Children’s Hospital, Seoul, South Korea; Department of Pediatrics, Seoul National University College of Medicine, Seoul, South Korea; Department of Pediatrics, Seoul National University Children’s Hospital, Seoul, South Korea; Department of Pediatrics, Pusan National University Yangsan Children’s Hospital and School of Medicine, Yangsan, South Korea; Department of Pediatrics, Korea University Guro Hospital, Seoul, South Korea; Department of Pediatrics, Kyungpook National University, School of Medicine, Daegu, South Korea; Department of Pediatrics, Chung-Ang University Hospital, Chung-Ang University College of Medicine, Seoul, South Korea; Department of Pediatrics, Hanyang University College of Medicine, Changwon Hanmaeum Hospital, Changwon, South Korea; Department of Pediatrics, Kyungpook National University, School of Medicine, Daegu, South Korea; Department of Pediatrics, Kyungpook National University, School of Medicine, Daegu, South Korea; Department of Pediatrics, Chonnam National University Hospital and Medical school, Gwangju, South Korea; Department of Pediatrics, Seoul National University College of Medicine, Seoul, South Korea; Department of Pediatrics, Seoul National University Children’s Hospital, Seoul, South Korea; Kidney Research Institute, Seoul National University Medical Research Center, Seoul, South Korea; Department of Pediatrics, Seoul National University College of Medicine, Seoul, South Korea; Department of Pediatrics, Seoul National University Children’s Hospital, Seoul, South Korea; Kidney Research Institute, Seoul National University Medical Research Center, Seoul, South Korea

**Keywords:** chronic kidney disease, growth rate, longitudinal studies, nephrogenic diabetes insipidus, pediatrics

## Abstract

**Background:**

Primary nephrogenic diabetes insipidus (NDI) is a rare inherited disorder with limited data on long-term outcomes. This study assessed longitudinal outcomes with primary NDI.

**Methods:**

This multicenter retrospective study included 63 patients with primary NDI. Growth rates and estimated glomerular filtration (eGFR) were analyzed via a piecewise linear mixed-effects model during the pediatric period.

**Results:**

*AVPR2* and *AQP2* mutations were identified in 74.6% and 9.5% of patients, respectively. The median ages at diagnosis and last follow up were 0.41 and 12.37 years, respectively. Height *Z*-scores declined from birth to 1.5 years and improved thereafter, with slopes of −2.626, −0.563, 0.102, and 0.031 per year across breakpoints at 0.5, 1.5, and 7 years. Although none of the patients met the criteria for short stature by age 18, the mean final height *Z*-score remained below average and was significantly lower than that at birth. Weight and weight-for-height *Z*-scores initially declined and then improved, with slope changes at 0.5 and 0.7 years. By the age of 18, 38.9% were overweight or obese. eGFR increased rapidly before 1.4 years (slope 25.61 ml/min/1.73 m^2^/year), and slowly thereafter (slope 0.58), reaching eGFR ≥90 ml/min/1.73 m^2^ at a median age of 5.1 years. The proportion of patients with eGFR <90 ml/min/1.73 m^2^ decreased to 34.6% at 14 years, then rose to 43.8% by the age of 18. Treatment-associated complications included hypokalemia (64.5%), alkalosis (61.1%), and hyperuricemia (60.7%).

**Conclusions:**

Patients with primary NDI exhibit a dynamic growth trajectory, delayed achievement of normal GFR with subsequent risk of chronic kidney disease, and frequent treatment-associated complications. These findings underscore the need for timely and individualized long-term management.

## Graphical Abstract

**Graphical Abstract:**
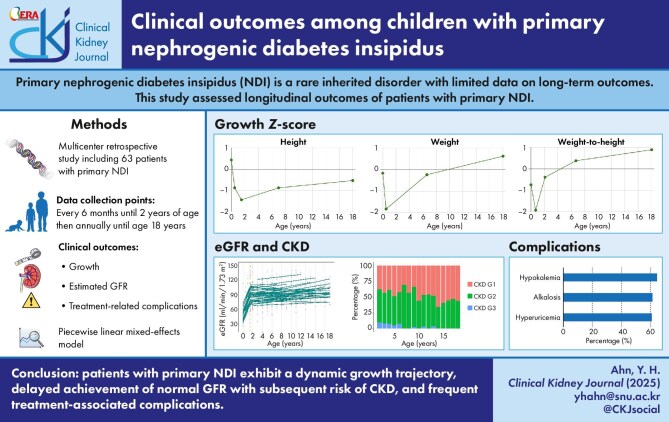

KEY LEARNING POINTS **What was known:**

- Primary nephrogenic diabetes insipidus (NDI) is a rare inherited disease characterized by impaired urinary concentration, leading to dehydration, electrolyte imbalances, failure to thrive, growth retardation, and chronic kidney disease (CKD) beginning in infancy.

**This study adds:**

- Patients with primary NDI exhibit a dynamic growth trajectory, characterized by early decline followed by gradual improvement, alongside a rising prevalence of obesity during adolescence.
- Treatment-related complications, such as hypokalemia, alkalosis, and hyperuricemia, were frequently observed.
- Although eGFR gradually increased during early childhood, the prevalence of CKD rose again during adolescence.

**Potential impact:**

- This multicenter longitudinal study enhances understanding of the long-term clinical course of primary NDI, helping predict outcomes and inform management strategies.
- Ongoing monitoring of growth, kidney function, and medication side-effects is essential to guide individualized care and improve long-term outcomes in this vulnerable population.

## INTRODUCTION

Primary nephrogenic diabetes insipidus (NDI), a rare inherited disorder, impairs arginine-vasopressin signaling in the renal collecting ducts, causing an inability to concentrate urine [1]. Most cases are attributed to mutations in two key genes: arginine-vasopressin receptor 2 (*AVPR2*) and aquaporin-2 (*AQP2*) genes [1, 2]. These genetic defects lead to severe polyuria and compensatory polydipsia placing patients, especially infants, at risk of dehydration, fever, and failure to thrive.

The estimated incidence of primary NDI is ∼1 in 100 000 [1]. Although several studies have described growth retardation, developmental delays, mental health issues, and urological complications in affected childhood [3–7], few have assessed long-term clinical trajectories. A recent European cohort study reported increased rates of obesity and chronic kidney disease (CKD) in NDI patients during adulthood [6]. However, few studies have evaluated longitudinal changes in growth rate and kidney function from childhood through adolescence and into adulthood in patients with NDI.

Treatment with diuretics and non-steroidal anti-inflammatory drugs reduce urine volume but may risk hypokalemia, hyperuricemia, and gastrointestinal bleeding [4, 6]. These treatment-associated complications [8], have not been systematically evaluated in long-term pediatric follow up.

This study evaluates longitudinal outcomes in NDI patients, analyzing serial changes in growth patterns, treatment-related complications, and kidney function over time.

## MATERIALS AND METHODS

### Study design

This multicenter retrospective study included children diagnosed with primary NDI at seven pediatric nephrology centers between 1985 and 2023. Inclusion criteria were as follows: (i) genetically confirmed pathogenic variants in the *AVPR2* or *AQP2* gene; or (ii) a clinical NDI confirmed by a water deprivation test and desmopressin test [9]. Patients with secondary causes of NDI, including drug-induced, autoimmune diseases, hypercalcemia, and cystic kidney disease, were excluded. Medical records were retrospectively reviewed; body measurements, laboratory findings, and medication details were collected at intervals of 6 months until the age of 2 years and then annually. Data collected within the first 3 months were categorized as 0 year. This study was approved by the Institutional Review Boards of all participating centers, which waived the requirement for informed consent due to the retrospective nature of the study.

### Definitions

Height, weight, and body mass index (BMI) *Z*-scores were calculated using the 2017 Korean National Growth Charts (KNGC2017) for children and adolescents [10]. Short stature was defined as a height *Z*-score <−1.88; BMI categories for children aged >2 years: underweight (BMI *Z*-score, <−1.65), normal (−1.65 to <1.04), overweight (1.04 to <1.65), and obesity (≥1.65) [10]. For children <2 years of age, weight-for-length *Z*-scores were calculated using the World Health Organization growth charts [11]. To analyze changes in weight-for-length and BMI *Z*-scores collectively over time, they were referred to as weight-relative-to-height *Z*-scores. Hypokalemia was defined as a serum potassium level <3.5 mmol/l. Alkalosis was defined as a serum total CO_2_ level >30 mmol/l [12]. Hyperuricemia was defined as a serum uric acid level >6.8 mg/dl [13]. CKD stages G2 and G3 were defined by an estimated glomerular filtration rate (eGFR) of 60–<90 and 30–<60 ml/min/1.73 m^2^, respectively, according to the 2024 Kidney Disease: Improving Global Outcomes guidelines [14]. The eGFR was calculated using the creatinine-based Chronic Kidney Disease in Children under 25 (CKiD U25) formula for children, adolescents, and young adults [15]. Ultrasonographic findings were assessed for hydronephrosis severity using the Onen grading system [16]. Bladder wall thickening was defined as >3 mm in a distended bladder or >5 mm in a non-distended bladder [17]. Developmental delay was defined as a failure to attain age-appropriate developmental milestones in one or more domains in children under 5 years of age, based on DSM-5 criteria [18]. Psychiatric disorders were defined as conditions diagnosed by a psychiatrist according to DSM-5 diagnostic criteria.

### Statistical analysis

Data are expressed as numbers (percentages) for categorical variables and medians with interquartile ranges (IQR) for continuous variables. A linear mixed model analyzed growth, electrolytes, and eGFR changes with age, reporting estimated marginal means (EMM) with 95% confidence intervals (CI). Segmented regression identified breakpoints in growth trajectories for height, weight, and weight-relative-to-height, as well as eGFR changes. Breakpoints were estimated by fitting a segmented regression model using the “segmented” package in R, allowing detection of points where the slope of the growth curve significantly changed [19]. Following breakpoint identification, a piecewise linear mixed-effects model was employed to assess growth rates and eGFR changes across distinct time intervals. The mixed-effects model was implemented using the “lmer” function from the ‘lme4’ package in R [20], and *t*-tests were conducted using the Satterthwaite’s method via the “lmerTest” package [21]. The number of breakpoints was determined by goodness-of-fit statistics [22]. The Akaike information criterion, Bayesian information criterion, and log-likelihood were used to determine the optimal number of breakpoints for the selection criteria. A Kaplan–Meier survival analysis evaluated time to eGFR ≥90 ml/min/1.73 m^2^. Generalized linear mixed model assessed longitudinal changes in the prevalence of CKD ≥G2 over age. For genotype–phenotype correlation analysis, patients with *AVPR2* mutations were categorized into two groups: (i) the predicted loss-of-function (pLoF) variant group, which included nonsense, frameshift, splice site, and copy number variants; and (ii) the non-pLoF group, which included missense, in-frame deletion, and in-frame duplication variants. To minimize the risk of false positives arising from multiple comparisons across time points in growth, electrolyte, and eGFR analyses between the two groups, Bonferroni correction was applied as *post hoc* adjustment. Categorical variables were analyzed using the Fisher’s exact test, whereas continuous variables were compared using the Mann–Whitney *U*-test. Statistical analyses were performed using R version 4.2.2. A *P* value <.05 was considered statistically significant for all tests.

## RESULTS

### Patient characteristics

Sixty-three patients with primary NDI from 53 families were included (Table 1). Median ages at diagnosis and last follow up were 0.41 (IQR, 0.12–1.28) and 12.37 (IQR, 7.34–19.61) years, respectively.

**Table 1:** Baseline characteristics.

Characteristics	Total (*n* = 63)
Sex, male:female	59:4
Causative gene, *n* (%)	
*AVPR2*	47 (74.6)
*AQP2*	6 (9.5)
Not identified	6 (9.5)
Not done	4 (6.3)
Characteristics at diagnosis	
Onset age, years	0.41 (0.12 to 1.28)
Height *Z*-score	−0.575 (−1.685 to 1.116)
Weight *Z*-score	−0.722 (−2.417 to 0.521)
BMI, kg/m^2^	14.26 (13.47 to 15.72)
Weight to height *Z*-score	−1.267 (−2.384 to −0.042)
eGFR, ml/min/1.73 m^2^	57.59 (45.66 to 70.36)
Serum sodium, mmol/l	152 (145 to 158)
Serum potassium, mmol/l	4.6 (4.3 to 4.8)
Serum chloride, mmol/l	117 (111 to 122)
Serum osmolarity, mOsm/kg	314 (300 to 325)
Urine osmolarity, mOsm/kg	105 (77 to 145)
Treatment, *n* (%)	
Thiazide	63 (100.0)
Potassium-sparing diuretics	57 (90.5)
Indomethacin	23 (36.5)
Potassium chloride	17 (27.0)
Uric acid-lowering agents	11 (17.5)
Growth hormone	6 (9.5)
Age at last follow up, years	12.37 (7.34 to 19.61)
eGFR at last follow up, ml/min/1.73 m^2^	90.81 (73.84 to 106.62)
Drug related complications, *n* (%)	
Hypokalemia	40/62 (64.5)
Alkalosis	33/54 (61.1)
Hyperuricemia	34/56 (60.7)
Disease related complications, *n* (%)	
Bladder dysfunction	6 (9.5)
Developmental delay	14 (22.2)
Psychiatric problems	11 (17.5)

Values are presented as numbers (%) or median (interquartile ranges).

### Genetic diagnosis

Pathogenic variants in *AVPR2* and *AQP2* were identified in 47 (74.6%) and 6 (9.5%) patients, respectively (Supplementary  Table S1). No pathogenic variants were detected in six patients, and four did not undergo genetic testing. Single nucleotide variants in *AVPR2* included missense (*n* = 29), frameshift (*n* = 4), nonsense (*n* = 2), and in-frame deletion (*n* = 1) variants. Eleven patients had copy number variants in *AVPR2*. Among patients with *AQR2* variants, all but one exhibited autosomal recessive inheritance. A single patient had an autosomal dominant *AQP2* variant, characterized by a de novo frameshift variant in the C terminus of *AQP2*.

### Growth

The EMM for height *Z*-scores declined from 0.540 (95% CI, 0.207 to 0.873) at 0 year to −1.324 (95% CI, −1.629 to −1.019) at 1.5 years, before improving to −0.823 (95% CI, −1.206 to −0.440) by 18 years of age (*P* < .001) (Supplementary Table S2). *Post hoc* analysis revealed a significant difference in height Z-scores between 0 and 18 years of age (*P* < .001).

**Figure 1:**
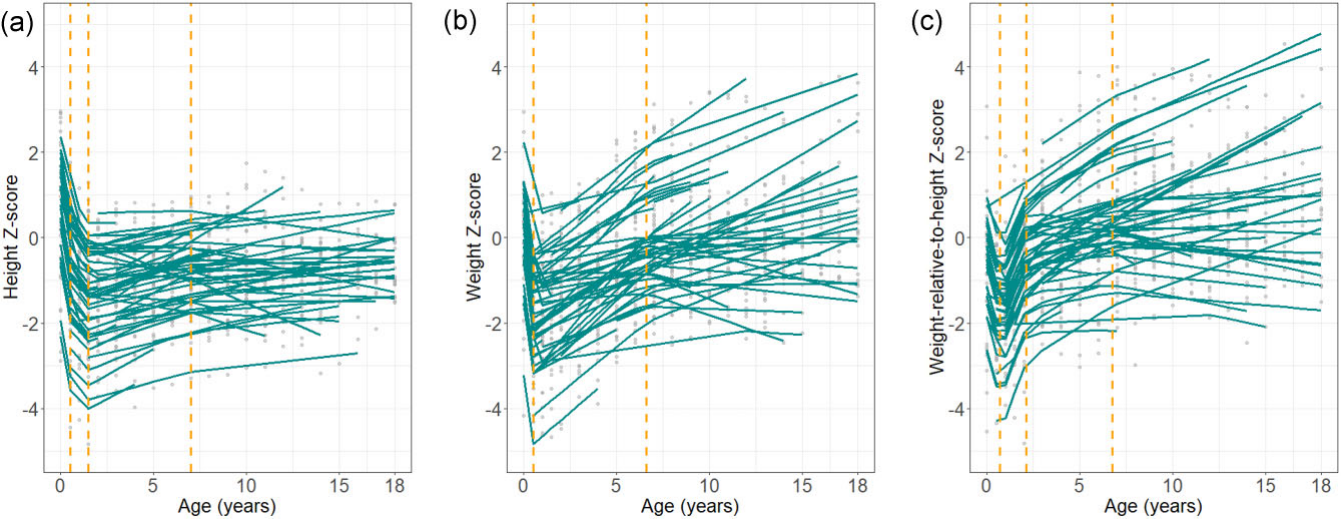
Longitudinal patterns in growth parameter *Z*-scores fitted models and identified breakpoints. (**a**) Height *Z*-scores with breakpoints at 0.5, 1.5, and 7.0 years of age. (**b**) Weight *Z*-scores with breakpoints at 0.5 and 6.6 years of age. (**c**) Weight-relative-to-height *Z*-scores with breakpoints at 0.7, 2.1, and 6.7 years of age.

A piecewise linear mixed-effects model identified key growth phases, with slopes of −2.616, −0.563, 0.102, and 0.031 per year across the identified breakpoints (Fig. 1a, *P* < .001 in all phases except *P* = .001 after the third breakpoint). The number of breakpoints was determined using goodness-of-fit statistics (Supplementary Table S3). The proportion of short stature increased to 39.0% (16 of 41 patients) at 1.5 years, and declined to 0% (0 of 8 patients) by 18 years (Supplementary Fig. S1a). Six patients initiated growth hormone therapy at a median age of 11.68 years (IQR, 9.36–12.91 years), due to growth hormone deficiency (*n* = 3), reduced growth velocity (*n* = 2), or height *Z*-scores below −2.5 (*n* = 1). At treatment initiation, the median height *Z*-score was −1.879 (IQR, −2.508 to −1.430), which improved to −0.978 (IQR, −1.151 to −0.030) at the last follow up (median age 13.31 years; IQR, 11.03–15.16 years) (*P* = .031, Wilcoxon signed-rank test).

The EMM for weight *Z*-scores decreased from 0.034 (95% CI, −0.369 to 0.437) at 0 year to −1.716 (95% CI, −2.083 to −1.350) at 1 year, and then increased to 0.434 (95% CI, −0.029 to 0.897) at 18 years (*P* < .001). Two breakpoints were identified, with slopes of −3.339, 0.261, and 0.076 per year across the phases (Fig. 1b, *P* < .001 in all phases).

For weight-relative-to-height *Z*-scores, the EMM decreased from −0.625 (95% CI, −1.065 to −0.184) at 0 year to −1.482 (95% CI, −1.882 to −1.082) at 1 year, followed by a steady increase to 1.039 (95% CI, 0.526 to 1.551) by 18 years (*P* < .001). Three breakpoints were identified, with slopes of −1.701, 1.057, 0.167, and 0.045 per year across the phases (Fig. 1c, *P* < .001 in all phases). Although the proportion of underweight decreased from 22.6% at 2 years to 0% at 18 years, the proportion of overweight and obesity increased from 6.3% at 2 years to 38.9% at 18 years (Supplementary Fig. S1b).

**Figure 2:**
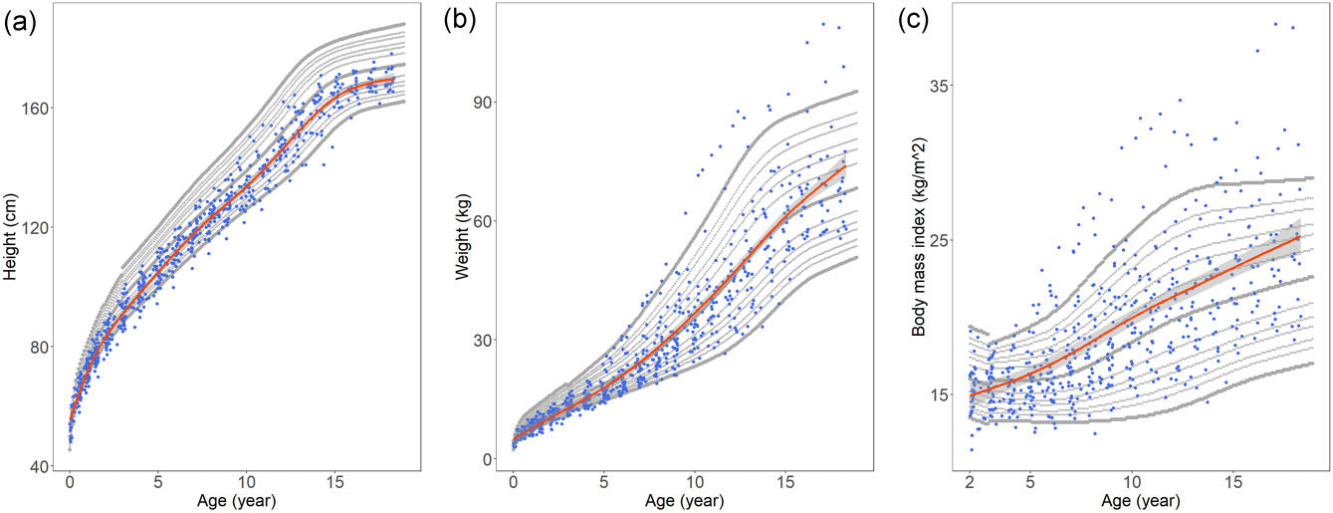
Scatter plots of height (**a**), weight (**b**), and BMI (**c**) for male patients with primary nephrogenic diabetes. Gray dots represent data from the 2017 Korean National Growth Charts, with percentile lines displayed for the 1st, 3rd, 5th, 10th, 15th, 25th, 50th, 75th, 85th, 90th, 95th, 97th, and 99th percentiles. Bold dots indicate the 1st, 50th, and 99th percentiles. Red lines indicate trend lines for the patients’ growth parameters.

Figure 2 illustrates the growth trends in male patients with primary NDI compared to the Korean growth charts for the general population [10]. Height remained consistently lower than that of the general population across all ages, whereas weight and BMI exceeded the average trajectories.

### Treatment and complications

Thiazide treatment was administered to all the patients, with 57 (90.5%) also receiving potassium-sparing agents and 23 (36.5%) receiving indomethacin (Table 1 and Supplementary Fig. S2). Five patients, including one with an autosomal dominant *AQP2* variant, discontinued medication at a median 6.37 years (IQR, 6.26–12.09 years). Compared to those who continued treatment, these patients were more often female, were diagnosed at a later age, and had lower sodium and chloride levels at diagnosis (Supplementary Table S4). At last follow up, they showed higher serum sodium and chloride levels than those who discontinued medication.

Thiazide dose per kilogram decreased over time (Supplementary  Table S5). The serum potassium levels decreased over time, whereas the total CO_2_ and uric acid levels increased (Supplementary Table S6). Hypokalemia occurred in 40/62 (64.5%) at a median 3.96 years (IQR, 2.34–7.40 years), and 17 patients received potassium chloride. No patients experienced hypokalemia-associated symptoms such as muscle weakness, numbness, and vomiting. Alkalosis developed in 33/54 (61.1%) at a median 7.38 years (IQR, 4.36–10.13 years). Hyperuricemia was diagnosed in 34/56 (60.7%) at a median 10.58 years (IQR, 4.57–14.04 years); 11 received uric acid-lowering agents such as allopurinol, benzbromarone, and febuxostat. Two patients with hyperuricemia developed gout at 16.36 and 27.36 years of age.

Bladder function disorders, including daytime enuresis and dysfunctional voiding, occurred in six (9.5%) patients. Developmental delays were observed in 14 (22.2%) patients. Psychiatric problems were identified in 11 (17.5%) patients, including attention-deficit hyperactivity disorder (*n* = 7), intellectual disability (*n* = 5), and tic disorder (*n* = 3).

Ultrasonographic data in 56 (88.9%) showed hydronephrosis (42.8%), distal ureter dilatation (25.0%), bladder wall thickening (19.6%), and increased cortical echogenicity (7.1%). All cases of hydronephrosis were classified as Onen grade 1.

### Changes in eGFR

**Figure 3:**
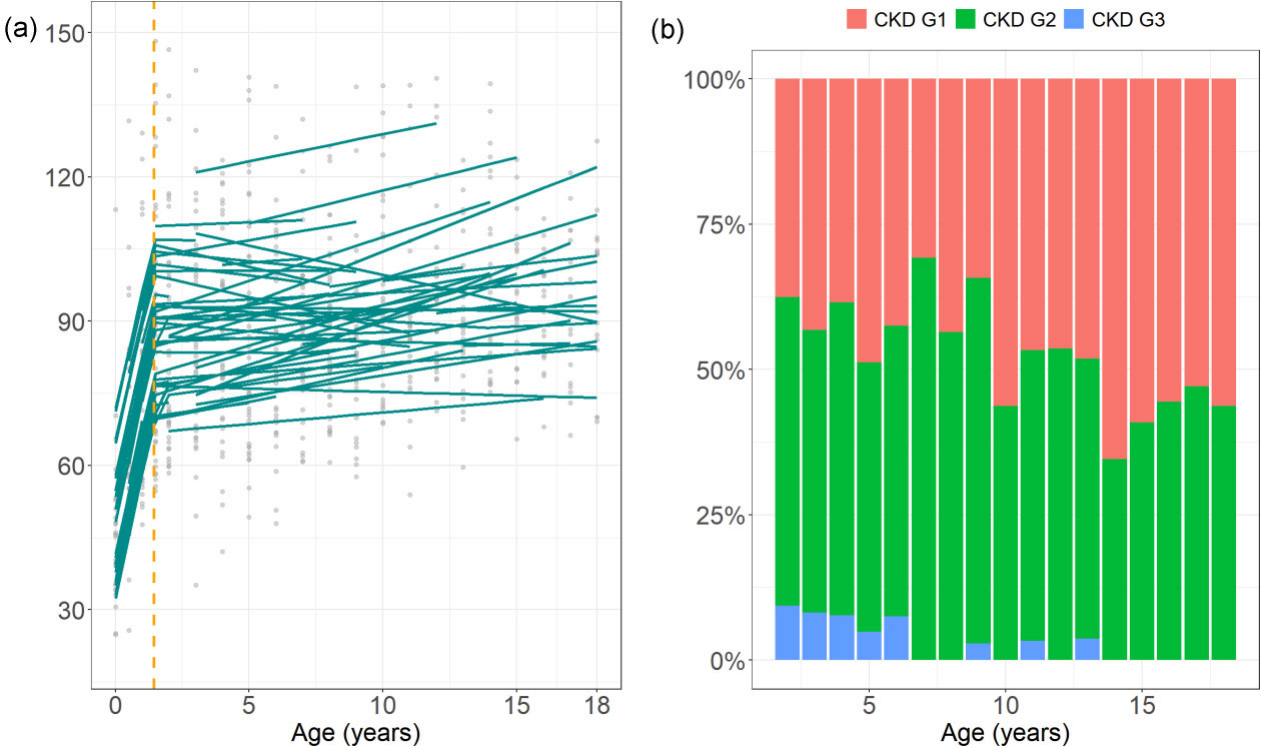
Longitudinal changes in eGFR. (**a**) Longitudinal eGFR changes with a fitted model and breakpoint at 1.4 years of age, as determined by a piecewise linear mixed-effects model. (**b**) Proportion of patients with CKD G1, 2, and 3 across different ages.

The EMM for eGFR increased steadily from 46.6 ml/min/1.73 m^2^ at 0 year to 96.9 ml/min/1.73 m^2^ by 18 years of age (*P* < .001) (Supplementary Table S6). A piecewise linear mixed-effects model identified distinct phases, with slopes of 25.61 ml/min/1.73 m^2^ per year before 1.43 years of age and 0.58 ml/min/1.73 m^2^ per year thereafter (Fig. 3a, *P* < .001). The Kaplan–Meier analysis showed that the median time to achieve an eGFR of 90 ml/min/1.73 m^2^ was 5.13 years (95% CI, 3.01–8.02). The prevalence of CKD ≥G2 decreased from 62.5% at age 2 years to 34.6% at age 14 years, and subsequently increased to 43.8% by the age 18 years (Fig. 3b). A generalized linear mixed model showed that the odds of CKD ≥G2 were significantly lower at age 14 years compared with that at 2 years of age, with an estimated coefficient of −1.547 (*P* = .021). At the last follow up, 44.4% of patients had an eGFR <90 ml/min/1.73 m^2^, although no patient had an eGFR <60 ml/min/1.73 m^2^. The change in eGFR and time to achieve an eGFR of 90 ml/min/1.73 m^2^ did not differ with the use of indomethacin (Supplementary Fig. S3).

### Genotype–phenotype correlation

**Figure 4:**
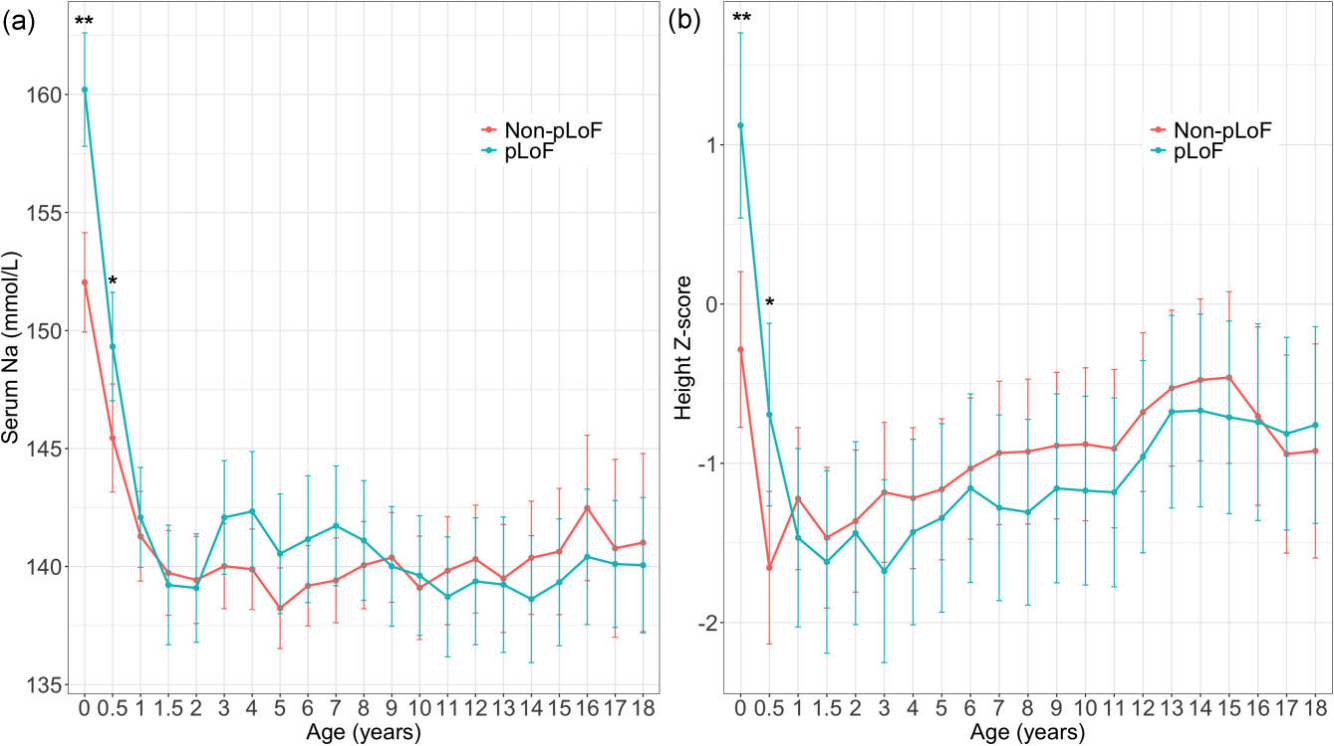
Changes in (**a**) sodium levels and (**b**) height *Z*-scores over time between non-pLoF and pLoF groups. The EMM over time are presented, and the vertical bars represent the 95% CI. *P < .001 between the non-pLoF and pLoF groups *P < .05 between the non-pLoF and pLoF groups. pLoF, predicted loss-of-function.

Genotype–phenotype correlation analysis was conducted in patients with *AVPR2* variants. Baseline characteristics at diagnosis were comparable between the non-pLoF (*n* = 30) and pLoF (*n* = 17) groups, except for serum sodium and chloride levels (Table 2). A linear mixed-effects model revealed significant differences in the trajectory of serum sodium levels with age between the two groups (*P* < .011) (Fig. 4a). At 0 and 0.5 years of age, serum sodium levels were significantly higher in the pLoF group than those in the non-pLoF group (*post hoc* analysis, *P* < .001 and *P* = 019, respectively). A linear mixed-effects model demonstrated a higher height *Z*-score at birth in the pLoF group. However, this group exhibited a more rapid decline in height *Z*-scores over time than did the non-pLoF group (*P* < .001) (Fig. 4b). Although height *Z*-scores at 18 years of age in the non-pLoF group did not differ from those at birth, the pLoF group showed a significant decline between birth and 18 years of age (*P* = .004). No significant differences were observed between the groups with respect to other growth parameters over time. In addition, the changes in eGFR and time to achieve an eGFR of 90 ml/min/1.73 m^2^ were similar between the two groups.

**Table 2:** Comparison of clinical characteristics in patients with *AVPR2* mutations according to mutation types.

Characteristics	Non-pLoF (*n* = 30)	pLoF (*n* = 17)	*P* value
Sex, male:female	30:0	17:0	1.000
Characteristics at diagnosis			
Onset age, years	0.43 (0.11 to 1.23)	0.18 (0.05 to 0.54)	.219
Height *Z*-score	−1.435 (−1.753 to 0.670)	0.061 (−1.658 to 1.680	.258
Weight *Z*-score	−0.957 (−2.493 to 0.375)	−0.061 (−3.115 to 0.574)	.728
BMI, kg/m^2^	14.11 (13.74 to 15.89)	13.48 (13.11 to 14.69)	.376
Weight to height *Z*-score	−0.750 (−2.384 to 0.066)	−1.165 (−2.763 to 0.027)	.823
eGFR at diagnosis, ml/min/1.73 m^2^	57.59 (47.74 to 66.08)	45.73 (32.36 to 70.58)	.107
Serum Na, mmol/l	149.0 (145.0 to 155.0)	159.0 (152.0 to 161.5)	.004
Serum K, mmol/l	4.6 (4.2 to 4.7)	4.7 (4.5 to 4.9)	.202
Serum Cl, mmol/l	115.0 (111.0 to 118.0)	121.5 (117.0 to 127.0)	.008
Serum osmolarity, mOsm/kg	312.0 (300.0 to 320.0)	322.5 (312.0 to 339.5)	.092
Urine osmolarity, mOsm/kg	104.5 (86.0 to 149.0)	118.5 (88.5 to 412.5)	.540
Treatment, *n* (%)			
Thiazide	30 (100.0)	17 (100.0)	1.000
Potassium-sparing diuretics	28 (93.3)	14 (82.4)	.336
Indomethacin	12 (40.0)	4 (23.5)	.343
Potassium chloride	8 (26.7)	5 (29.4)	1.000
Uric acid-lowering agents	6 (20.0)	2 (11.8)	.692
Growth hormone	3 (10.0)	1 (5.9)	1.000
Age at last follow up, years	9.25 (6.14 to 15.24)	18.34 (4.77 to 23.91)	.147
eGFR at last follow up, ml/min/1.73 m^2^	87.74 (69.70 to 100.34)	90.81 (72.50 to 98.32)	.826
Drug related complications, *n* (%)			
Alkalosis	15/23 (65.2)	9/16 (56.3)	.740
Hypokalemia	20/29 (69.0)	9/17 (52.9)	.349
Hyperuricemia	14/25 (56.0)	11/16 (68.8)	.519
Disease related complications, *n* (%)			
Bladder dysfunction	1 (3.3)	2 (11.8)	.544
Developmental delay	5 (16.7)	4 (23.5)	.704
Psychiatric problems	7 (23.3)	3 (17.6)	.727

Values are presented as numbers (%) or median (interquartile ranges).

## DISCUSSION

This multicenter study enabled a comprehensive longitudinal analysis of clinical outcomes in patients with primary NDI, providing novel insights into growth trajectories, treatment-related complications, and kidney function changes. Our findings revealed that patients with NDI experience a dynamic and evolving trajectory in terms of growth, marked by periods of rapid decline followed by improvement over time, and a rising prevalence of obesity. Treatment-related complications, including hypokalemia, alkalosis, and hyperuricemia, were frequently observed. Furthermore, while eGFR gradually increased during childhood, the prevalence of CKD rose in adolescence.

Patients with primary NDI have a biphasic growth pattern, with a steep decline in early childhood, followed by a slower recovery in later years. The proportion of patients with short stature peaked at 39.0% at 1.5 years but declined to 0% by 18 years. This early vulnerability underscores the critical need for timely intervention, including pharmacologic treatment, adequate caloric intake, tube feeding in cases of recurrent vomiting and dehydration, and dietary counseling particularly during infancy [9]. Notably, the transition from a negative to a positive growth slope in the height *Z*-score occurred later than that in the weight *Z*-score, suggesting that nutritional status plays a significant role in these differences. Despite the improvement, the mean final height *Z*-score remained below the average within normal limits and was significantly lower than the height *Z*-score at birth, indicating that patients with NDI may not have fully achieved their growth potential. In our cohort, 9.5% of patients received growth hormone therapy due to growth hormone deficiency, reduced growth velocity, or height *Z*-scores below −2.5. All treated patients exhibited subsequent improvements in height *Z*-scores. Although growth hormone therapy is not routinely recommended for growth impairment in primary NDI, it may be considered in selected cases with confirmed growth hormone deficiency or inadequate growth despite optimal management [23].

The weight-relative-to-height *Z*-scores declined early, then increased, with overweight/obesity rising to 38.9% by age 18, while underweight vanished. A previous study reported a high prevalence of obesity in adults with primary NDI [6]; however, our findings indicate that obesity may begin as early as childhood and adolescence. Even after improvements in weight gain, continued intake of a high-calorie diet may have contributed to the development of obesity in these patients. The widening distribution of BMI with age (Fig. 2c) suggests considerable inter-individual variability in weight trajectories. Clinicians should continuously monitor growth patterns and nutritional status, providing timely and individualized dietary interventions in collaboration with pediatric dietitians [24]. While early nutritional support is essential during infancy to ensure adequate growth, appropriate calorie management becomes increasingly important during childhood and adolescence to prevent obesity.

Medication-related complications were common in this cohort. Hypokalemia (64.5%), alkalosis (61.1%), and hyperuricemia (60.7%) occurred with diuretic use. Although no hypokalemia-related symptoms were observed, hypokalemia could be associated with life-threatening complications such as arrhythmia, even in asymptomatic cases. Long-term use of thiazides frequently leads to hypokalemia, even when co-administered with potassium-sparing agents. Although 90.5% of the patients were on potassium-sparing agents, 27% received potassium supplementation. Indomethacin could be effective in reducing the side-effects of diuretics and improving patients’ quality of life by controlling polyuria; however, its long-term use carries the risk of CKD and gastrointestinal complications [2, 8]. In our cohort, indomethacin use was not associated with significant changes in eGFR trajectories. Moreover, no patients receiving indomethacin experienced gastrointestinal adverse events such as abdominal pain, vomiting, or gastrointestinal bleeding. Although hyperuricemia has not been a primary focus in previous studies, it was prevalent in this cohort and is likely associated with prolonged thiazide use and obesity. Increasing levels of uric acid with age may be associated with prolonged thiazide use and increasing prevalence of obesity in this population. Although rare, some patients in this cohort developed gout due to hyperuricemia. Individualized regimens balancing benefits and side-effects, including electrolyte disturbances, are crucial. Regular monitoring and proactive management of these complications are essential to optimize patient outcomes. In line with this, recent expert consensus guidelines recommend regular blood testing of sodium, potassium, chloride, bicarbonate, creatinine, and uric acid to assess treatment efficacy and monitor for adverse effects [9].

eGFR rose rapidly before 1.43 years, then slowly, with a median time of 5.1 years to achieve an eGFR ≥90 ml/min/1.73 m^2^. These findings differ from those of the general population, where the eGFR increases rapidly, peaking at ∼104 ml/min/1.73 m^2^ around 2 years of age and stabilizing thereafter [25]. At the last follow up, 44.4% of the patients had an eGFR <90 ml/min/1.73 m^2^, consistent with the findings in previous studies [4–6]. The prevalence of CKD ≥G2 in this cohort decreased with age, reaching its lowest level at 14 years, after which it increased. Recurrent dehydration episodes may contribute to a slower increase in eGFR and CKD development in these patients. Epidemiologic data support an association between inadequate hydration and an increased risk of CKD [26–30]. An experimental study demonstrated that chronic recurrent dehydration, combined with periodic water intake, exacerbated kidney function decline, hypertension, kidney inflammation, and fibrosis in rats [31]. A large-scale cohort study reported that 32% of children with primary NDI had CKD ≥G2, which increased to 48% in adulthood [6]. This study also demonstrated that eGFR declines with age in adults, with the prevalence of CKD being significantly higher in patients with NDI than in the general population [6]. Therefore, long-term monitoring of kidney function in patients with primary NDI is crucial, as the eGFR increases slowly during childhood and may decline again in adulthood.

Previous studies have reported no significant differences in clinical parameters—such as age at diagnosis, growth, kidney function, or complications—between patients with *AVPR2* and *AQP2* mutations [5, 6]. This study demonstrated that patients with pLoF mutations in the *AVPR2* gene exhibited higher serum sodium and chloride levels and experienced a more rapid decline in height Z-scores during infancy. Similarly, a European cohort study showed that patients with pLoF mutations are diagnosed earlier than those with missense mutations [6]. These results indicate that severe causative mutations in the *AVPR2* gene may lead to more pronounced clinical manifestations in patients with NDI. Although the initial difference in birth length between the pLoF and non-pLoF groups reached statistical significance, it may reflect other influences such as parental height or selection bias. Unfortunately, parental height data were not available in our cohort, limiting our ability to adjust for this potential confounder.

This retrospective cohort study had several limitations. First, the availability of data was limited, as patients were not evaluated according to a standardized protocol with scheduled follow ups. Second, kidney function assessment was constrained by the use of a creatinine-based eGFR formula. The recent KDIGO guidelines recommended calculating eGFR using both serum creatinine and cystatin C. However, as many patients in this study were diagnosed and followed during a period when cystatin C testing was not routinely available, an eGFR formula incorporating cystatin C could not be applied in the analysis. Third, interpretation of growth patterns may be limited, as data on nutritional intake and supplementation affecting growth were not incorporated in the analysis. In addition, cultural factors in South Korea contribute to caregivers’ reluctance toward tube feeding and gastrostomy, which contrast with previous studies reporting that 20%–30% of children with primary NDI received tube feeding at some point [4–6]. Despite these limitations, this study is a multicenter cohort study with a relatively large sample size for this rare disease, offering valuable longitudinal follow-up data on clinical outcomes. The findings provide important insights into growth patterns and kidney function trajectories in patients with NDI.

In conclusion, this study highlighted the complex and evolving clinical course of primary NDI, which is characterized by early growth retardation followed by catch-up growth, delayed achievement of normal GFR with subsequent risk of CKD, and a high prevalence of treatment-related complications. The early decline in height and weight *Z*-scores, followed by an increased prevalence of obesity throughout childhood and adolescence, underscores the need for continuous, age-appropriate nutritional interventions. Clinicians must remain vigilant in monitoring growth trajectories, kidney function, and treatment-related side-effects, thereby enabling timely and individualized care—including proactive medication adjustments and tailored nutritional support—that addresses both the short- and long-term challenges faced by patients with primary NDI.

## Supplementary Material

sfaf303_Supplemental_File

## Data Availability

We are pleased to share data supporting this study upon reasonable request to the corresponding author.
