# Supplementary material for: Clinical outcomes among children with primary nephrogenic diabetes insipidus
Source: Clin Kidney J. 2025 Oct 1;18(11):sfaf303. doi: 10.1093/ckj/sfaf303 (PMC12585523; doi:10.1093/ckj/sfaf303)

**Supplementary Materials**

**Table S1:** Genetic diagnosis of patients with primary nephrogenic diabetes insipidus.

**Table S2:** Growth parameters according to age.

**Table S3:** Results of goodness-of-fit statistics for the better model fit.

**Table S4:** Comparison of clinical characteristics between patients continuing and discontinuing medications

**Table ~~S4~~ S5:** Doses and usage of medications according to age.

**Table ~~S5~~ S6:** Estimated glomerular filtration rate and electrolytes according to age.

**Figure 1:** (a) Proportion of patients with short stature across different ages. (b) Proportion of body mass index categories across different ages.

**Figure 2:** Trends in medications usage over time.

**Figure 3:** (a) Longitudinal changes in estimated glomerular filtration rate (eGFR) over time based on indomethacin usage. Estimated marginal means are presented, with vertical bars indicating the 95% confidence intervals. (b) Cumulative incidence of achieving an eGFR ≥90 mL/min/m^2^, as estimated by Kaplan-Meier analysis, stratified by indomethacin usage.

**Table S1. Genetic diagnosis of patients with primary nephrogenic diabetes insipidus.**

| Patient ID | Family ID | Gene | cDNA change | Protein change | ACMG criteria | Pathogenicity |
| --- | --- | --- | --- | --- | --- | --- |
| 1 | 1 | *AVPR2* | *AVPR2* disruption due to 4q25 insertional translocation | NA | NA | LP |
| 2 | 1 | *AVPR2* | *AVPR2* disruption due to 4q25 insertional translocation | NA | NA | LP |
| 3 | 1 | *AVPR2* | *AVPR2* disruption due to 4q25 insertional translocation | NA | NA | LP |
| 4 | 2 | *AVPR2* | Total deletion | NA | NA | P |
| 5 | 3 | *AVPR2* | c.337C>T | p.Arg113Trp | PM1,PM2,PM5,PP3,PP5 | P |
| 6 | 3 | *AVPR2* | c.337C>T | p.Arg113Trp | PM1,PM2,PM5,PP3,PP5 | P |
| 7 | 4 | Not identified | NA | NA | NA | NA |
| 8 | 5 | *AVPR2* | c.965C>A | p.Pro322His | PM1,PM2,PM5,PP3 | P |
| 9 | 5 | *AVPR2* | c.965C>A | p.Pro322His | PM1,PM2,PM5,PP3 | P |
| 10 | 6 | *AVPR2* | Partial deletion (downstream of exon 1) | NA | NA | P |
| 11 | 7 | *AVPR2* | Total deletion | NA | NA | P |
| 12 | 8 | *AVPR2* | c.490T>C | p.Trp164Arg | PM1,PM2,PM5,PP3,PP5 | P |
| 13 | 9 | *AVPR2* | c.526_528del | p.Phe176del | PM1,PM2,PM4,BP4 | LP |
| 14 | 10 | *AVPR2* | Total deletion | NA | NA | P |
| 15 | 10 | *AVPR2* | Total deletion | NA | NA | P |
| 16 | 11 | *AVPR2* | c.245G>T | p.Cys82Phe | PM1,PM2,PP3 | LP |
| 17 | 12 | *AVPR2* | c.410G>A | p.Arg137His | PM1,PM2,PP3,PP5 | P |
| 18 | 13 | *AQP2* | c.559C>T  c.374C>T | p.Arg187Cys  p.Thr125Met | PM1,PM2,PM3,PM5,PP3,PP5  PM2,PM3,PP2,PP3,PP5 | P  P |
| 19 | 14 | *AVPR2* | c.967T>C | p.Trp432Arg | PM1,PM2,PP3 | LP |
| 20 | 15 | *AVPR2* | c.965C>A | p.Pro322His | PM1,PM2,PM5,PP3 | P |
| 21 | 16 | *AQP2* | c.789del | p.Ser264AlafsTer71 | PVS1,PM2 | LP |
| 22 | 17 | *AVPR2* | c.383A>C | p.Tyr128Ser | PM1,PM2,PP3,PP5 | LP |
| 23 | 18 | *AVPR2* | Total deletion | NA | NA | P |
| 24 | 19 | Not identified | NA | NA | NA | NA |
| 25 | 20 | *AVPR2* | Deletion for approximately 196 bases of exon 2 | NA | NA | P |
| 26 | 21 | Not identified | NA | NA | NA | NA |
| 27 | 22 | *AVPR2* | c.500C>T | p.Ser167Leu | PM1,PM2,PP3,PP5 | LP |
| 28 | 23 | *AVPR2* | c.500C>T | p.Ser167Leu | PM1,PM2,PP3,PP5 | LP |
| 29 | 24 | *AVPR2* | c.500C>T | p.Ser167Leu | PM1,PM2,PP3,PP5 | LP |
| 30 | 25 | *AVPR2* | c.251C>A | p.Ala84Asp | PM1,PM2,PP3 | LP |
| 31 | 25 | Not identified | NA | NA | NA | NA |
| 32 | 26 | *AVPR2* | c.541C>T | p.Arg181Cys | PM1,PM2,PP3,PP5 | P |
| 33 | 27 | *AVPR2* | c.1009C>T | p.Arg337Ter | PVS1,PM2,PP5 | P |
| 34 | 27 | *AVPR2* | c.1009C>T | p.Arg337Ter | PVS1,PM2,PP5 | P |
| 35 | 28 | *AVPR2* | c.143_144del | p.Phe48CysfsTer143 | PVS1,PM2 | LP |
| 36 | 29 | *AQP2* | c.127_128del  c.190G>A | p.Gln43AspfsTer63  p.Gly64Arg | PVS1,PM2,PM3,PP5  PS1,PM1,PM2,PM3,PP3,PP5 | P  P |
| 37 | 30 | *AQP2* | c.209C>A  c.560G>A | p.Ala70Asp  p.Arg187His | PM1,PM2,PM3,PP3,PP5  PM1,PM2,PM3,PM5,PP3,PP5 | P  P |
| 38 | 31 | Not done | NA | NA | NA | NA |
| 39 | 32 | *AVPR2* | c.604C>T | p.Arg202Cys | PM1,PM2,PP5 | P |
| 40 | 33 | Not done | NA | NA | NA | NA |
| 41 | 34 | Not done | NA | NA | NA | NA |
| 42 | 35 | *AVPR2* | c.604C>T | p.Arg202Cys | PM1,PM2,PP5 | P |
| 43 | 36 | Not identified | NA | NA | NA | NA |
| 44 | 37 | *AVPR2* | c.292G>C | p.Ala98Pro | PM1,PM2,PP1,PP3 | LP |
| 45 | 37 | *AVPR2* | c.292G>C | p.Ala98Pro | PM1,PM2,PP1,PP3 | LP |
| 46 | 38 | *AVPR2* | c.620C>A | p.Thr207Asn | PM1,PM2,PP3 | LP |
| 47 | 39 | *AVPR2* | Total deletion | NA | NA | P |
| 48 | 40 | *AVPR2* | c.314T>C | p.Phe105Ser | PM1,PM5,PM2,PP3 | LP |
| 49 | 41 | *AVPR2* | c.410G>A | p.Arg137His | PM1,PM2,PM5,PP3,PP5 | P |
| 50 | 41 | *AVPR2* | c.410G>A | p.Arg137His | PM1,PM2,PM5,PP3,PP5 | P |
| 51 | 42 | *AVPR2* | c.409del | p.Arg137AlafsTer25 | PV2,PM2 | LP |
| 52 | 43 | *AVPR2* | c.337C>T | p.Arg113Trp | PM1,PM2,PM5,PP3,PP5 | P |
| 53 | 44 | *AVPR2* | c.738del | p.Arg247AlafsTer24 | PVS1,PM2,PP5 | P |
| 54 | 45 | *AQP2* | c.127_128del  c.323C>T | p.Gln43AspfsTer63  p.Thr108Met | PVS1,PP5,PM2  PM2,PP2,PP3,PP5 | P  LP |
| 55 | 46 | *AVPR2* | c.427del | p.Arg143ValfsTer19 | PV1,PM2 | LP |
| 56 | 47 | *AVPR2* | c.500C>T | p.Ser167Leu | PM1,PM2,PP3,PP5 | LP |
| 57 | 48 | Not identified | NA | NA | NA | NA |
| 58 | 49 | *AQP2* | c.127_128del  c.116C>G | p.Gln43AspfsTer63  p.Pro39Arg | PVS1,PM2,PM3,PP5  PM2,PM3,PP2,PP3 | P  LP |
| 59 | 50 | *AVPR2* | c.410G>A | p.Arg137His | PM1,PM2,PM5,PP3,PP5 | P |
| 60 | 51 | *AVPR2* | c.575G>C | p.Cys192Ser | PM2,PP3,PP5 | LP |
| 61 | 52 | Not done | NA | NA | NA | NA |
| 62 | 53 | *AVPR2* | c.604C>T | p.Arg202Cys | PM1,PM2,PP5 | LP |
| 63 | 53 | *AVPR2* | c.604C>T | p.Arg202Cys | PM1,PM2,PP5 | LP |

ID, identification; ACMG, American College of Medical Genetics; NA, not available; LP, Likely pathogenic; P, pathogenic; VUS, variant of uncertain significance.

*Pathogenicity according to ACMG guideline: pathogenic criterion; very strong (PVS1), strong (PS1-4), moderate (PM1-6), or supporting (PP1-5), and benign criterion; strong (BS1-4), or supporting (BP1-6).

**Table S2. Growth parameters according to age.**

| Age  (years) | n | Height Z-score | Weight Z-score | Weight-relative-to-height  Z-score |
| --- | --- | --- | --- | --- |
| 0 | 34 | 0.540 (0.207 to 0.873) | 0.034 (-0.369 to 0.437) | -0.625 (-1.065 to -0.184) |
| 0.5 | 35 | -0.825 (-1.147 to -0.504) | -1.621 (-2.007 to -1.235) | -1.350 (-1.774 to -0.926) |
| 1 | 43 | -1.128 (-1.434 to -0.823) | -1.716 (-2.083 to -1.350) | -1.482 (-1.882 to -1.082) |
| 1.5 | 41 | -1.324 (-1.629 to -1.019) | -1.370 (-1.738 to -1.001) | -0.989 (-1.388 to -0.589) |
| 2 | 41 | -1.311 (-1.616 to -1.005) | -1.153 (-1.522 to -0.784) | -0.554 (-0.954 to -0.154) |
| 3 | 43 | -1.348 (-1.651 to -1.046) | -1.051 (-1.416 to -0.685) | -0.089 (-0.484 to 0.307) |
| 4 | 43 | -1.228 (-1.53 to -0.925) | -1.037 (-1.402 to -0.671) | -0.186 (-0.582 to 0.210) |
| 5 | 44 | -1.152 (-1.453 to -0.851) | -0.690 (-1.054 to -0.327) | 0.115 (-0.278 to 0.509) |
| 6 | 43 | -1.031 (-1.333 to -0.728) | -0.365 (-0.731 to 0.000) | 0.383 (-0.013 to 0.778) |
| 7 | 42 | -0.928 (-1.232 to -0.623) | -0.249 (-0.616 to 0.119) | 0.409 (0.011 to 0.807) |
| 8 | 43 | -0.959 (-1.263 to -0.655) | -0.210 (-0.578 to 0.157) | 0.411 (0.012 to 0.809) |
| 9 | 38 | -0.927 (-1.239 to -0.616) | -0.102 (-0.478 to 0.274) | 0.488 (0.080 to 0.897) |
| 10 | 35 | -0.849 (-1.166 to -0.531) | 0.035 (-0.349 to 0.419) | 0.629 (0.212 to 1.047) |
| 11 | 31 | -0.897 (-1.224 to -0.569) | 0.100 (-0.295 to 0.496) | 0.734 (0.302 to 1.166) |
| 12 | 29 | -0.767 (-1.100 to -0.434) | 0.077 (-0.326 to 0.48) | 0.627 (0.186 to 1.067) |
| 13 | 29 | -0.672 (-1.006 to -0.339) | 0.166 (-0.237 to 0.569) | 0.677 (0.235 to 1.118) |
| 14 | 27 | -0.631 (-0.971 to -0.292) | 0.314 (-0.096 to 0.725) | 0.779 (0.329 to 1.229) |
| 15 | 23 | -0.600 (-0.956 to -0.245) | 0.412 (-0.018 to 0.842) | 0.844 (0.371 to 1.317) |
| 16 | 20 | -0.718 (-1.088 to -0.348) | 0.593 (0.145 to 1.041) | 1.109 (0.614 to 1.603) |
| 17 | 19 | -0.819 (-1.195 to -0.442) | 0.519 (0.063 to 0.975) | 1.122 (0.618 to 1.626) |
| 18 | 18 | -0.823 (-1.206 to -0.441) | 0.434 (-0.029 to 0.897) | 1.039 (0.526 to 1.551) |

Z-scores are presented as estimated means with 95% confidence intervals, calculated using linear mixed model analysis.

**Table S3. Results of goodness-of-fit statistics for the better model fit.**

|  | Number of breakpoints | AIC | BIC | Log-likelihood |
| --- | --- | --- | --- | --- |
| Height Z-score | 0 | 1652 | 1679 | -820 |
|  | 1 | 1448 | 1480 | -717 |
|  | 2 | 1443 | 1479 | -713 |
|  | 3 | 1433 | 1474 | -707 |
| Weight Z-score | 0 | 1770 | 1798 | -879 |
|  | 1 | 1642 | 1674 | -814 |
|  | 2 | 1579 | 1615 | -781 |
|  | 3 | 1582 | 1623 | -782 |
| Weight-relative-to-height Z-score | 0 | 1964 | 1991 | -976 |
|  | 1 | 1899 | 1931 | -942 |
|  | 2 | 1870 | 1906 | -927 |
|  | 3 | 1844 | 1885 | -913 |
| eGFR | 0 | 5834 | 5861 | -2911 |
|  | 1 | 5741 | 5773 | -2864 |
|  | 2 | 5767 | 5803 | -2876 |
|  | 3 | 5770 | 5810 | -2876 |
| Thiazide dose | 0 | 1337 | 1365 | -663 |
|  | 1 | 1314 | 1346 | -650 |
|  | 2 | 1320 | 1356 | -652 |
|  | 3 | 1322 | 1363 | -652 |

Lower values of AIC and BIC indicate a better model fit. The higher the log-likelihood, the better the fit.

Akaike Information Criterion; BIC, Bayesian Information Criterion; LL, Log-likelihood; eGFR, estimated glomerular filtration rate.

**Supplementary Table S4:** Comparison of clinical characteristics between patients continuing and discontinuing medications

| **Characteristics** | **Continuing (n=58)** | **Discontinuing (n=5)** | ***P* value** |
| --- | --- | --- | --- |
| Sex, male:female | 56:2 | 3:2 | 0.029 |
| Causative gene, n (%) |  |  |  |
| *AVPR2* | 45 (77.6) | 2 (40.0) | 0.049 |
| *AQP2* | 4 (6.9) | 2 (40.0) |  |
| Not identified | 6 (10.3) | 0 (0.0) |  |
| Not done | 3 (5.2) | 1 (20.0) |  |
| At diagnosis |  |  |  |
| Onset age, years | 0.34 (0.11 to 1.02) | 2.54 (1.42 to 4.45) | 0.011 |
| Height Z-score | -0.58 (-1.71 to 1.12) | -0.57 (-1.16 to 0.09) | 0.483 |
| Weight Z-score | -0.99 (-2.49 to 0.47) | -0.44 (-0.53 to 0.54) | 0.292 |
| Weight to height Z-score | -1.47 (-2.40 to 0.03) | -0.66 (-0.91 to -0.41) | 0.564 |
| eGFR at diagnosis, mL/min/m^2^ | 52.84 (42.99 to 69.85) | 69.94 (59.14 to 88.78) | 0.107 |
| Serum Na, mmol/L | 152.0 (145.3 to 158.8) | 139.0 (137.0 to 144.5) | 0.047 |
| Serum K, mmol/L | 4.6 (4.3 to 4.8) | 4.8 (4.2 to 5.0) | 0.763 |
| Serum Cl, mmol/L | 118.0 (113.0 to 122.0) | 104.0 (103.0 to 109.5) | 0.032 |
| Serum osmolarity, mOsm/kg | 316.0 (302.0 to 325.5) | 301.5 (295.3 to 307.8) | 0.474 |
| Urine osmolarity, mOsm/kg | 106.5 (78.5 to 144.3) | 56.0 (47.0 to 89.0) | 0.114 |
| Treatment, n (%) |  |  |  |
| Thiazide | 58 (100.0) | 5 (100.0) | 1.000 |
| Potassium-sparing diuretics | 53 (91.4) | 4 (80.0) | 0.404 |
| Indomethacin | 22 (37.9) | 1 (20.0) | 0.644 |
| Potassium chloride | 17 (29.3) | 0 (0.0) | 0.312 |
| Uric acid lowering agents | 11 (19.0) | 0 (0.0) | 0.576 |
| Growth hormone | 6 (10.3) | 0 (0.0) | 1.000 |
| At stopping medications |  |  |  |
| Age, years | NA | 6.37 (6.36 to 12.09) | NA |
| eGFR at diagnosis, mL/min/m^2^ | NA | 80.28 (71.56 to 87.66) | NA |
| Serum Na, mmol/L | NA | 140.0 (139.0 to 142.0) | NA |
| Serum K, mmol/L | NA | 4.0 (4.0 to 4.3) | NA |
| Serum Cl, mmol/L | NA | 105.0 (101.0 to 107.0) | NA |
| Serum osmolarity, mOsm/kg | NA | 289.0 (288.0 to 290.0) | NA |
| At last follow-up |  |  |  |
| Age, years | 12.53 (7.23 to 19.15) | 10.49 (8.49 to 21.83) | 0.839 |
| eGFR, mL/min/m^2^ | 93.32 (73.50 to 106.88) | 86.21 (82.16 to 89.67) | 0.674 |
| Serum Na, mmol/L | 139.5 (138.0 to141.0) | 142.0 (140.0 to 144.0) | 0.035 |
| Serum K, mmol/L | 3.8 (3.6 to 4.1) | 3.8 (3.8 to 4.0) | 0.587 |
| Serum Cl, mmol/L | 102.0 (99.0 to 104.0) | 106.0 (105.0 to 107.0) | 0.001 |
| Serum osmolarity, mOsm/kg | 289.0 (285.0 to 291.0) | 290.0 (290.0 to 297.0) | 0.184 |
| Drug related complications, n (%) |  |  |  |
| Alkalosis | 31 (63.3) | 2/5 (40.0) | 0.336 |
| Hypokalemia | 38 (66.7) | 2/5 (40.0) | 0.337 |
| Hyperuricemia | 33 (63.5) | 1/4 (25.0) | 0.289 |

Values are presented as numbers (%) or median (interquartile ranges).

eGFR, estimated glomerular filtration rate; NA, not available.

**Table ~~S4~~ S5. Doses and usage of medications according to age.**

| Age  (years) | n | Thiazide dose  (mg/kg/day) | Thiazide  n (%) | K sparing diuretics  n (%) | Potassium chloride  n (%) | Indomethacin  n (%) | Uric acid lowering agents  n (%) |
| --- | --- | --- | --- | --- | --- | --- | --- |
| 0 | 34 | 1.33 (1.05 to 1.62) | 25 (75.8) | 14 (42.4) | 0 (0) | 2 (6.1) | 0 (0) |
| 0.5 | 35 | 1.40 (1.15 to 1.65) | 30 (85.7) | 17 (48.6) | 1 (2.9) | 4 (11.4) | 0 (0) |
| 1 | 43 | 1.53 (1.30 to 1.75) | 38 (88.4) | 23 (53.5) | 0 (0) | 3 (7.0) | 0 (0) |
| 1.5 | 41 | 1.82 (1.58 to 2.05) | 40 (97.6) | 29 (70.7) | 0 (0) | 4 (9.8) | 1 (2.4) |
| 2 | 41 | 1.73 (1.50 to 1.97) | 38 (95.0) | 27 (67.5) | 2 (5.0) | 4 (10.0) | 0 (0) |
| 3 | 43 | 1.54 (1.31 to 1.77) | 40 (95.2) | 26 (61.9) | 1 (2.4) | 5 (11.9) | 0 (0) |
| 4 | 43 | 1.60 (1.38 to 1.83) | 43 (100) | 29 (67.4) | 3 (7.0) | 5 (11.6) | 0 (0) |
| 5 | 44 | 1.63 (1.41 to 1.86) | 44 (100) | 32 (72.7) | 5 (11.4) | 8 (18.2) | 0 (0) |
| 6 | 43 | 1.52 (1.30 to 1.75) | 41 (95.4) | 32 (74.4) | 6 (14.0) | 8 (18.6) | 0 (0) |
| 7 | 42 | 1.56 (1.33 to 1.79) | 40 (95.2) | 31 (73.8) | 5 (11.9) | 9 (21.4) | 0 (0) |
| 8 | 43 | 1.50 (1.27 to 1.72) | 40 (93.0) | 34 (79.1) | 7 (16.3) | 8 (18.6) | 0 (0) |
| 9 | 38 | 1.47 (1.23 to 1.70) | 36 (94.7) | 31 (81.6) | 6 (15.8) | 7 (18.4) | 1 (2.6) |
| 10 | 35 | 1.31 (1.06 to 1.55) | 32 (91.4) | 27 (77.1) | 6 (17.1) | 6 (17.1) | 0 (0) |
| 11 | 31 | 1.35 (1.09 to 1.60) | 31 (100) | 25 (80.7) | 6 (19.4) | 3 (9.7) | 0 (0) |
| 12 | 29 | 1.29 (1.02 to 1.55) | 28 (96.6) | 23 (79.3) | 5 (17.2) | 5 (17.2) | 0 (0) |
| 13 | 29 | 1.20 (0.93 to 1.46) | 28 (96.6) | 24 (82.8) | 5 (17.2) | 4 (13.8) | 1 (3.5) |
| 14 | 27 | 1.10 (0.83 to 1.37) | 26 (96.3) | 22 (81.5) | 5 (18.5 | 4 (14.8) | 3 (11.1) |
| 15 | 23 | 1.08 (0.79 to 1.37) | 22 (95.7) | 19 (82.6) | 4 (17.4) | 3 (13.0) | 3 (13.0) |
| 16 | 20 | 1.04 (0.73 to 1.34) | 19 (95.0) | 16 (80.0) | 3 (15.0) | 2 (10.0) | 3 (15.0) |
| 17 | 19 | 0.93 (0.61 to 1.24) | 17 (89.5) | 15 (79.0) | 3 (15.8) | 1 (5.3) | 5 (26.3) |
| 18 | 18 | 0.91 (0.59 to 1.23) | 16 (88.9) | 14 (77.8) | 2 (11.1) | 0 (0) | 2 (11.1) |

Doses of thiazide are presented as estimated means with 95% confidence intervals, calculated using linear mixed model analysis.

**Table ~~S5~~ S6. Estimated glomerular filtration rate and electrolytes according to age.**

| Age  (years) | n | eGFR  (mL/min/m^2^) | Na  (mmol/L) | K  (mmol/L) | Cl  (mmol/L) | Total CO_2_  (mmol/L) | Uric acid  (mg/dL) |
| --- | --- | --- | --- | --- | --- | --- | --- |
| 0 | 34 | 46.6  (38.6 to 54.7) | 156  (154 to 157) | 4.86  (4.7 to 5.02) | 121  (119 to 123) | 23.1  (21.9 to 24.3) | 4.91  (4.29 to 5.53) |
| 0.5 | 35 | 66.8  (59.3 to 74.2) | 147  (146 to 149) | 4.40  (4.24 to 4.55) | 112  (110 to 114) | 23.9  (22.6 to 25.1) | 5.83  (5.22 to 6.44) |
| 1 | 43 | 75.3  (68.2 to 82.4) | 142  (140 to 143) | 4.21  (4.07 to 4.35) | 107  (105 to 108) | 23.9  (22.7 to 25.1) | 5.53  (4.94 to 6.12) |
| 1.5 | 41 | 89.9  (82.7 to 97) | 139  (138 to 141) | 4.13  (3.99 to 4.28) | 106  (104 to 107) | 24.4  (23.3 to 25.5) | 4.79  (4.21 to 5.38) |
| 2 | 41 | 85.8  (78.9 to 92.7) | 139  (138 to 141) | 3.93  (3.79 to 4.07) | 105  (103 to 106) | 25  (23.8 to 26.1) | 4.72  (4.12 to 5.32) |
| 3 | 43 | 85.2  (78.3 to 92.2) | 141  (139 to 142) | 3.81  (3.67 to 3.95) | 106  (104 to 107) | 24.4  (23.2 to 25.7) | 5.19  (4.59 to 5.79) |
| 4 | 43 | 86.2  (79.4 to 93.1) | 141  (139 to 142) | 3.85  (3.71 to 3.98) | 104  (103 to 106) | 26.3  (25.1 to 27.5) | 4.94  (4.34 to 5.53) |
| 5 | 44 | 89.2  (82.5 to 95.9) | 139  (138 to 140) | 3.84  (3.7 to 3.97) | 103  (101 to 104) | 26.3  (25.1 to 27.4) | 5.14  (4.55 to 5.72) |
| 6 | 43 | 88.8 (82 to 95.5) | 140  (138 to 141) | 3.8  (3.67 to 3.94) | 103  (101 to 104) | 27.1  (26 to 28.3) | 5.32  (4.73 to 5.92) |
| 7 | 42 | 84.2  (77.3 to 91) | 140  (139 to 141) | 3.89  (3.75 to 4.02) | 103  (102 to 105) | 27.6  (26.4 to 28.8) | 5.45  (4.89 to 6.02) |
| 8 | 43 | 87.2  (80.4 to 94) | 140  (139 to 141) | 3.86  (3.73 to 4) | 103  (101 to 104) | 28  (26.9 to 29.2) | 5.53  (4.95 to 6.12) |
| 9 | 38 | 86.9  (79.7 to 94) | 140  (138 to 141) | 3.87  (3.73 to 4.02) | 103  (101 to 104) | 28.5  (27.3 to 29.6) | 5.57  (4.97 to 6.17) |
| 10 | 35 | 98.1  (90.7 to 105.5) | 140  (138 to 141) | 3.82  (3.67 to 3.97) | 102  (101 to 104) | 28.1  (26.8 to 29.3) | 5.96  (5.34 to 6.58) |
| 11 | 31 | 90.6  (83 to 98.2) | 140  (138 to 141) | 3.85  (3.7 to 4.01) | 102  (100 to 103) | 28.2  (27 to 29.5) | 6.08  (5.46 to 6.71) |
| 12 | 29 | 93.3  (85.5 to 101.1) | 140  (139 to 142) | 3.80  (3.64 to 3.96) | 101  (99 to 103) | 28.6  (27.3 to 30) | 6.8  (6.15 to 7.44) |
| 13 | 29 | 91.6  (83.7 to 99.6) | 139  (138 to 141) | 3.82  (3.65 to 3.98) | 102  (100 to 104) | 28.3  (26.8 to 29.8) | 7.49  (6.81 to 8.18) |
| 14 | 27 | 99.7  (91.7 to 107.8) | 140  (138 to 141) | 3.71  (3.55 to 3.88) | 102  (100 to 104) | 30.5  (29 to 32) | 7.64  (6.99 to 8.29) |
| 15 | 23 | 95.5  (86.8 to 104.1) | 140  (138 to 141) | 3.81  (3.63 to 3.99) | 101  (99 to 103) | 29.4  (27.8 to 31) | 7.67  (6.97 to 8.38) |
| 16 | 20 | 94.1  (84.7 to 103.5) | 141  (139 to 143) | 3.79  (3.6 to 3.99) | 102  (100 to 104) | 29.6  (27.9 to 31.3) | 7.07  (6.38 to 7.77) |
| 17 | 19 | 93.9  (84.2 to 103.6) | 140  (138 to 142) | 3.88  (3.68 to 4.08) | 100  (98 to 102) | 29.5  (27.7 to 31.3) | 7.19  (6.46 to 7.91) |
| 18 | 18 | 96.9  (86.9 to 106.9) | 141  (139 to 143) | 3.78  (3.57 to 3.99) | 102  (100 to 104) | 29.4  (27.4 to 31.5) | 7.33  (6.61 to 8.05) |

Estimated GFR and electrolytes are presented as estimated means with 95% confidence intervals, calculated using linear mixed model analysis.

eGFR, estimated glomerular filtration rate.

**Figure S1:** (A) Proportion of patients with short stature across different ages. (B) Proportion of body mass index categories across different ages.


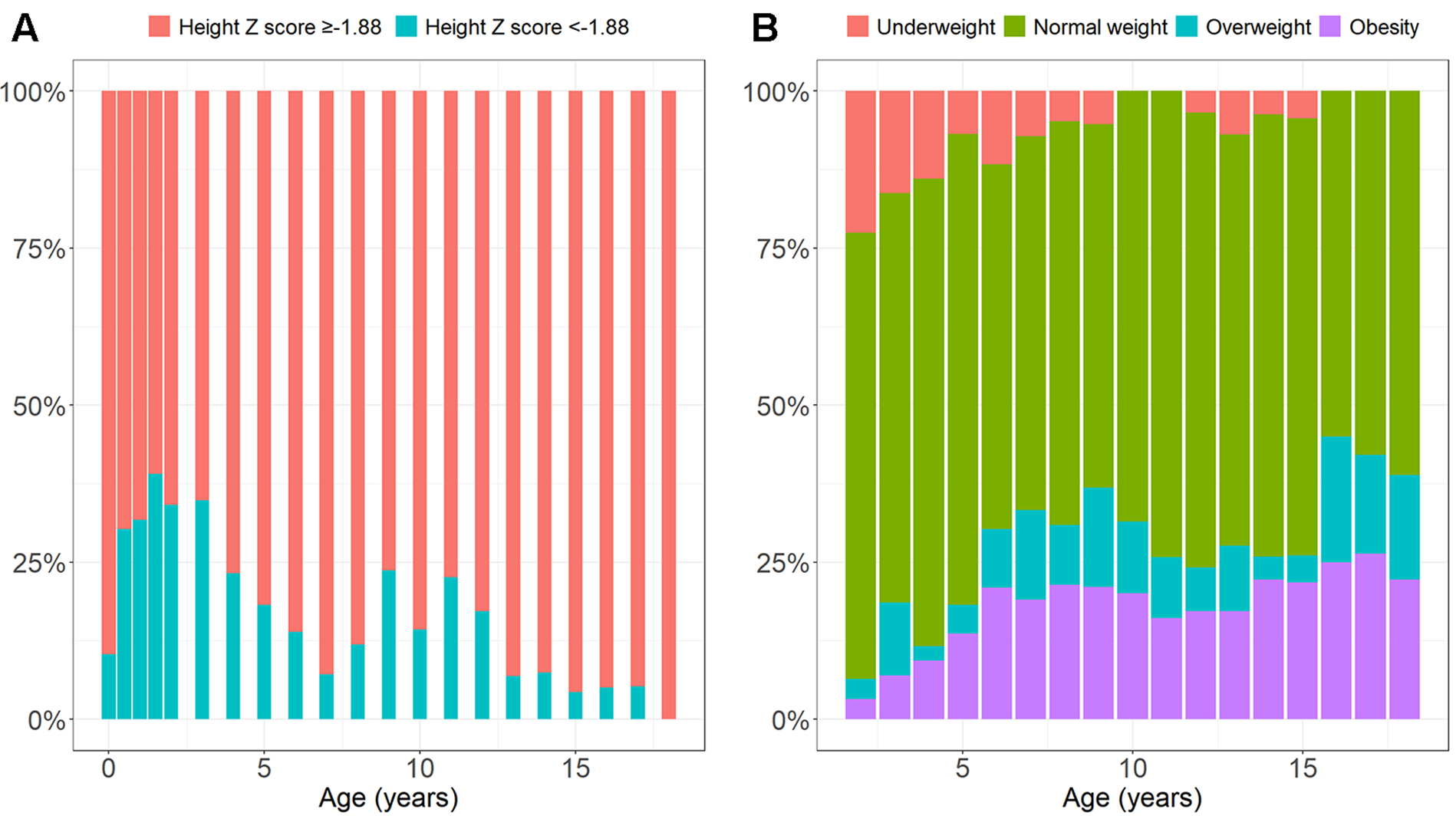


**Figure S2.** Trends in medications usage over time.


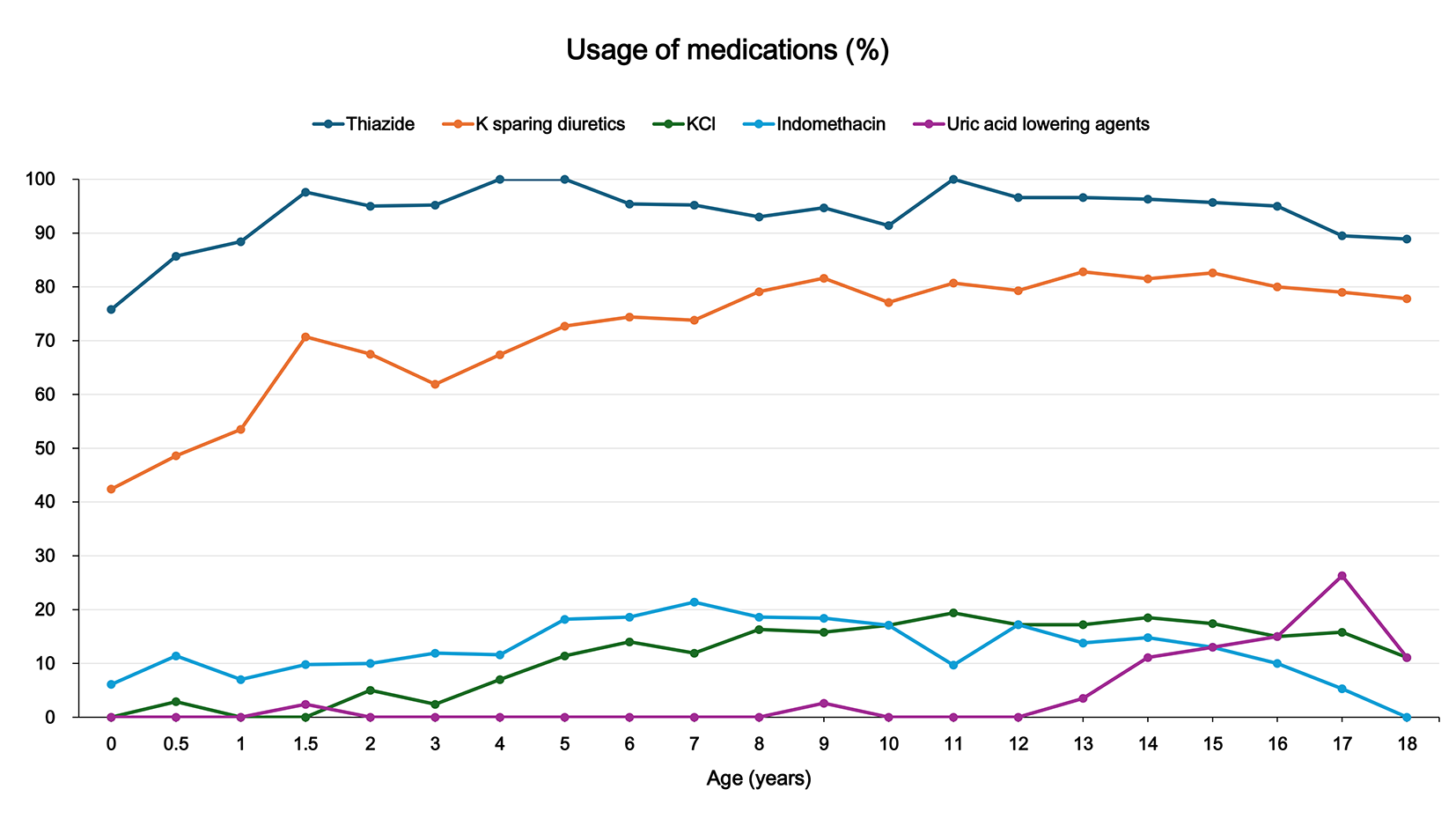


**Figure S3:** (A) Longitudinal changes in estimated glomerular filtration rate (eGFR) over time based on indomethacin usage. Estimated marginal means are presented, with vertical bars indicating the 95% confidence intervals. (B) Cumulative incidence of achieving an eGFR ≥90 mL/min/m^2^, as estimated by Kaplan-Meier analysis, stratified by indomethacin usage.


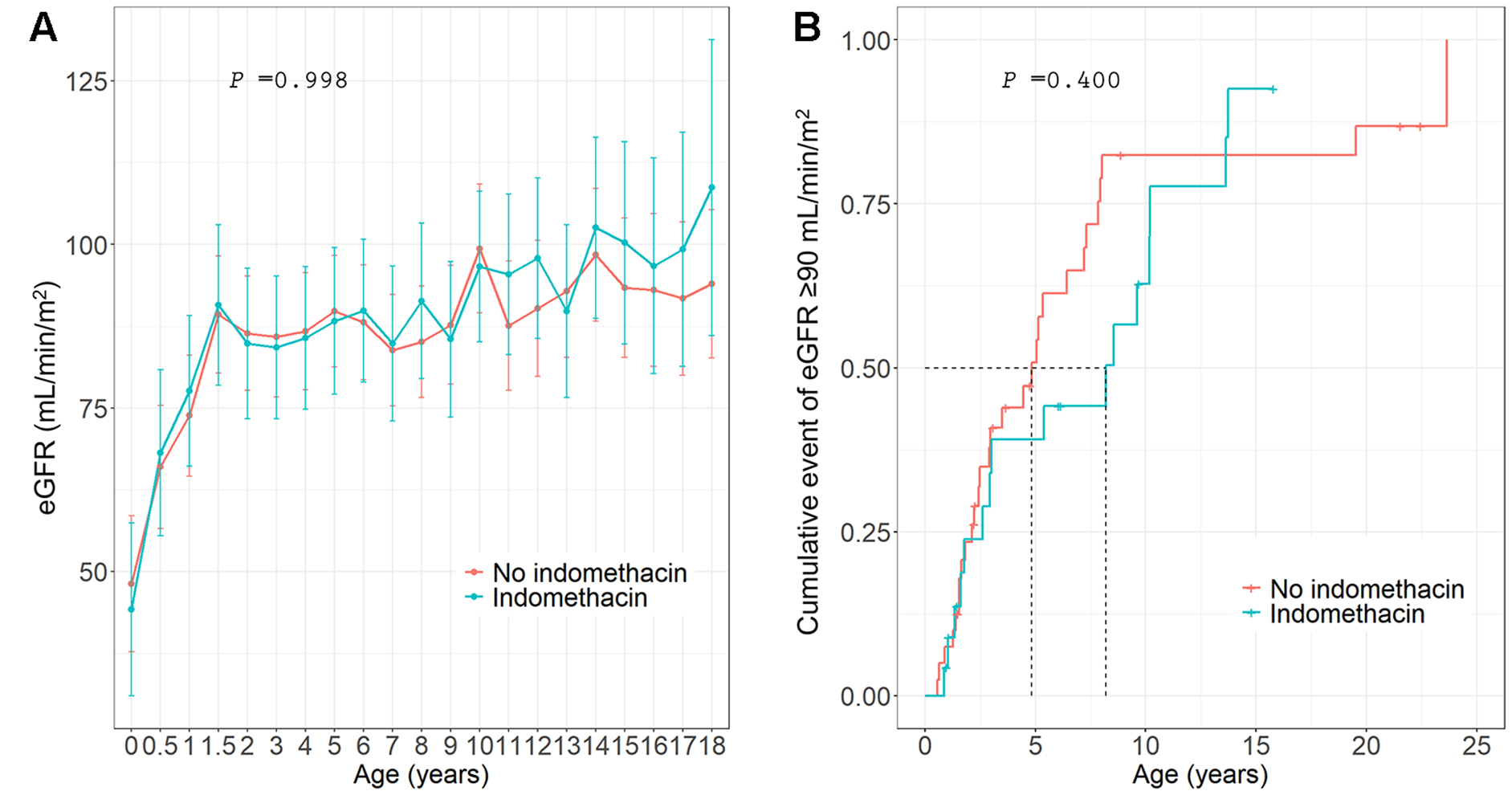

Supplement: sfaf303_Supplemental_File [file sfaf303_supplemental_file.docx]
